# Premenstrual Exacerbations of Mood Disorders: Findings and Knowledge Gaps

**DOI:** 10.1007/s11920-021-01286-0

**Published:** 2021-10-09

**Authors:** Christine Kuehner, Sibel Nayman

**Affiliations:** Research Group Longitudinal and Intervention Research, Department of Psychiatry and Psychotherapy, Medical Faculty Mannheim, Central Institute of Mental Health, University of Heidelberg, J5, 68159 Mannheim, Germany

**Keywords:** Premenstrual exacerbation, Menstrual cycle, Mood disorders, Depression, Bipolar disorder

## Abstract

**Purpose of Review:**

In contrast to premenstrual dysphoric disorder (PMDD), premenstrual exacerbations (PMEs) of ongoing mood disorders are understudied. The aim of this review is to describe diagnostic issues, epidemiology, underlying mechanisms, and treatment for PME in unipolar depression and bipolar disorder, and to discuss clinical and research implications.

**Recent Findings:**

Community-based and clinical studies estimate that in women with mood disorders around 60% report PME, while some women with bipolar disorder also show symptom exacerbations around ovulation. In general, PME predicts a more severe illness course and an increased burden. While heightened sensitivity to fluctuations of sex hormone levels across the menstrual cycle appears to contribute to PME and PMDD, the overlap of their underlying biological mechanisms remains unclear. Beneficial treatments for PMDD show less or no efficacy in PME. Pharmacological treatments for PME in mood disorders predominantly seem to profit from adjustable augmentation of treatment dosages during the luteal phase for the underlying disorder. However, the evidence is sparse and mainly based on earlier small studies and case reports.

**Summary:**

Previous research is mainly limited by the lack of a clear differentiation between PME and PMDD comorbidity with mood disorders. More systematic research with uniformly defined and prospectively assessed subgroups of PME in larger epidemiological and clinical samples is needed to receive reliable prevalence estimates and information on the clinical impact of PME of mood disorders, and to uncover underlying mechanisms. In addition, larger randomized controlled trials are warranted to identify efficacious pharmacological and psychotherapeutic treatments for affected women.

## Introduction

Reproductive hormones act on neurotransmitter systems and brain regions involved in mood regulation and emotional processing [1•, 2–5, 6•]. In a subgroup of women, reproductive transition periods such as the menstrual cycle, the peripartum, and the perimenopause are accompanied by significant changes in emotional, cognitive, and behavioral functioning [7–11].

In the context of premenstrual disorders (PMDs), the International Society for Premenstrual Disorders (ISPMD) [12] distinguishes between core PMDs, including premenstrual dysphoric disorder (PMDD) and severe premenstrual syndrome (PMS), and variants of PMD, including premenstrual exacerbations (PMEs) of ongoing mental or somatic disorders. Notably, while together with this differentiation the PMDD literature has grown consistently [13], there is a remarkable paucity of well-designed studies addressing epidemiology, possible underlying mechanisms, and treatment of PME of mood disorders. The aim of this review is to discuss diagnostic issues on the differentiation between PMDD and PME of depressive and bipolar disorders, to describe the available evidence on PME of mood disorders, and to discuss potential clinical and research implications.

## Diagnostic Issues

The typical menstrual cycle lasts about 28 (21 to 35) days. The follicular phase, lasting from menses to ovulation, is characterized by low progesterone (P4) and rising estrogen (E2) levels with a peak prior to ovulation, which is followed by a rapid E2 decrease after ovulation. During the luteal phase (days from ovulation until menses), E2 and P4 gradually rise, reaching their highest levels during the mid-follicular phase, and then show a rapid withdrawal during the late luteal (premenstrual) phase, i.e., the week prior to the next menses [14•].

### Differentiation of PMDD and PME of Mood Disorders

According to DSM-5 [15], PMDD is characterized by affective core symptoms (marked affective lability, irritability/anger/increased interpersonal conflicts, depressed mood/feelings of hopelessness, or anxiety/tension/feelings of being keyed up or on edge) and additional psychological and/or physical symptoms that are confined to the premenstrual phase and remit after the onset of menses.

In contrast to PMDD, PMEs of mood disorders manifest as a premenstrual worsening of an ongoing depressive or bipolar disorder (BD) [12, 16•]. A specific variant of PME is “premenstrual breakthrough,” describing a premenstrual entrainment (onset) of symptoms of the underlying condition, which is treatment-responsive during the rest of the cycle [17].

While the ISPMD definition requires to count each shared symptom of PME and PMDD towards PME, even if it represents a diagnostic criterion for PMDD (e.g., depressed mood) [12], DSM-5 does not clearly specify the differentiation of PMDD and PME in criterion E for PMDD: “The disturbance is not merely an exacerbation of the symptoms of another disorder … although it may co-occur with any of these disorders” [15]. The more conservative approach of the ISPMD [12, 18••, 19] aims to prevent prevalence overestimation for comorbid conditions and, importantly, possible inadequate treatment in women with improper dual diagnosis [19].

The differentiation of PMDD from PMEs requires prospective symptom ratings across at least two symptomatic menstrual cycles to consider the extent of postmenstrual symptoms [12]. However, while detailed instructions exist on how to identify the required pre- and postmenstrual symptom increases and decreases for PMDD with appropriate scales [20], there is no commonly accepted definition for respective rates of change to justify PME of another disorder. For depressive disorders, some authors require at least moderate postmenstrual symptom levels [19], but this definition does not take adequate account of premenstrual breakthroughs with largely symptom-free intervals during the rest of the cycle. To differentiate PME from PMDD, pre- and postmenstrual assessments should also include all symptoms of the underlying disorder, not only those of PMDD [19], which has been rarely done in related research.

The situation is even more complicated in BD. The Canadian Network for Mood and Anxiety Treatments (CANMAT) guidelines require for an accurate diagnosis of comorbid PMDD in women with BD that a stable state of euthymia must be reached during the remaining cycle phases, with a minimum of 2 months of prospective pre- and postmenstrual symptom charting [21, 22]. However, the vast majority of BD studies use non-mutually exclusive categories or do not control for ongoing mood episodes when studying comorbid PMDD [23, 24•].

### Comorbidity of PMDD and PME of Mood Disorders

Comorbidity of PMDD and PME of mood disorders can exist, but may be difficult to assess due to symptom overlaps. It characterizes a condition in which regularly premenstrual worsening of an ongoing disorder (e.g., depression) is accompanied by at least five non-overlapping PMDD symptoms (e.g., mood swings, anger, irritability, and physical symptoms) that occur only during the premenstrual phase [18••, 19]. Reliable estimates on the prevalence of respective comorbidities are lacking.

## Epidemiology, Risk Factors, and Consequences of PME in Mood Disorders

### PME in Depressive Disorders

Unipolar depression is a mood disorder with a high risk for recurrences or relapses and chronic developments. The gender ratio is around 2:1 (women:men), both in community-based and treatment settings [10]. Studies assessing the prevalence of PME of depressive disorders are rare, and more recent research is lacking. However, there is broad consensus that a majority of women seeking initial treatment for PMS or PMDD are in fact suffering from PME, mainly from PME of unipolar depression [16•, 25•]. Most affected women will therefore probably contact their gynecologists at first instance, although empirical data are lacking.

The only study that so far estimates prevalence rates of PME of depression from a representative community sample (*n* = 900 women aged 13–53) identified 58 women (6.4%) with a current depressive disorder and an unknown proportion of bipolar cases [26•]. Two-month prospective assessments of premenstrual symptoms with additional items for major depression (MDD) or dysthymia showed that 58% of the sample with a current depressive disorder exhibited PME of one or more depressive symptoms. PME, in turn, predicted decreased general functioning. More than 40% of women without current depression who took antidepressants also showed PME.

Payne et al. [27] asked women with MDD and BD (*n* > 2500) retrospectively whether they had ever noticed regular premenstrual or menstrual mood changes. In MDD patients, the respective prevalence rate was 68.5% compared to 33.7% in women with no diagnosis. PME symptoms significantly co-occurred with symptoms of other reproductive-related events (postpartum, perimenopausal) in the MDD group, but not in women with BD.

Two reports from the large multicenter Sequences Treatment Alternatives to Relieve Depression (STAR-D) study asked women with MDD whether they were aware of regularly occurring premenstrual mood worsening. Of 433 naturally menstruating women aged 18 to 61 years, 64% retrospectively reported PME of their depression [28]. In the full STAR-D sample of women with current MDD (*n* = 821; aged 18–39 years), the prevalence of retrospectively reported PME amounted 66% [29]. PME was linked to older age, longer index episodes, more depressive episodes in the past, a higher rate of familial history of both depressive disorders and BD, higher anxiety levels, more medical conditions, and poorer physical functioning. During the following treatment phase with citalopram, the time to relapse after remission was shorter for women with PME.

In a treatment study by Harvey et al. [30], chronic depressed patients were randomized to a 12-week sertraline or imipramine treatment plus crossover if patients were nonresponding. Premenstrual worsening of depression, anxiety, irritability, mood swings, and fatigue at baseline predicted higher rates of depressive worsenings during the follow-up assessments, irrespective of menstrual cycle phase and drug class. Depressive worsenings, in turn, predicted treatment nonresponses and dropouts.

### PME in Bipolar Disorder

Bipolar disorder (BD) is a recurrent chronic disorder characterized by fluctuations in mood state and energy, ranging from manic to depressive episodes [15]. Whereas the gender ratio for BD type I is equal, BD type II as well as rapid cycling, hypomania with mixed depressive phenotypes, and seasonal patterns of presentation are more prevalent in females [15, 31, 32].

A comprehensive review [24•] demonstrated that in retrospective studies 64–68% and in prospective studies 44–65% of women with BD reported menstrual cycle–related mood changes. Depressive, hypomanic, manic, or mixed symptoms are mainly entrained (i.e., likely to have their onset in) or exacerbated in the premenstrual phase, but also around ovulation and in the menstrual phase [24•]. A subgroup of women exhibits PME of depressive symptoms, while others report premenstrual or periovulatory exacerbation of hypomanic or manic symptoms, indicating that there may be a variety of symptom trajectories across the menstrual cycle in women with BD [24•].

In the longitudinal Systematic Treatment Enhancement Program for Bipolar Disorder (STEP-BD) [33•], 65.2% of 293 women with BD aged 18 to 40 years retrospectively reported PME of depressed or mood swing symptoms. During a 1-year follow-up period, self-reported PME of symptoms was linked to higher symptom severity, more depressive episodes, and mood elevation, regardless of cycle phase, and a faster time to relapse into syndromal or subsyndromal episodes. However, the STEP-BD project itself does not consequently distinguish PME from comorbid PMDD. In a more recent STEP-BD report on 1099 BD women, a comorbid PMDD diagnosis was assessed with a questionnaire covering the DSM-5 PMDD criteria. BD women with PMDD showed an earlier onset of BD with a smaller gap between BD onset and age of menarche, more comorbid Axis I disorders, a higher number of hypomanic/manic and depressive episodes, and higher rates of rapid cycling, which again points to worse clinical outcomes and increased burden of illness [34]. Since this study disregarded a possible overlap of PMDD symptoms with PME of BD, a substantial proportion of PMDD cases in this study may have fulfilled the criteria for PME according to the ISPMD definition [12]. Ignoring of PME may also explain the high rate of retrospectively identified rapid cycling in the comorbid PMDD group. According to prospective studies, PME in BD is not linked to rapid cycling [24•]. Several prospective small-scale studies or case reports failed to find consistent links between BD and the menstrual cycle [e.g., 23, 35, 36], indicating that not all women with BD experience PME or that appropriate treatment may counteract respective symptoms [37].

## Biological Mechanisms Underlying Premenstrual Exacerbations of Mood Disorders

The similar cyclical time pattern of PMDD and PME symptomatology in mood disorders appears to reflect a shared pathophysiology. However, this view has been increasingly questioned, and the overlap of underlying biological mechanisms in PMDD and PME remains unclear [25•, 38].

Nevertheless, there is broad consensus that both PMDD and PME of mood disorders do not arise from abnormal hormone levels, but that symptoms are triggered by abnormal sensitivity to normal fluctuations of sex hormones and their metabolites across the menstrual cycle [1•, 8, 18••, 24•, 39, 40]. Cycle-related fluctuations of sex hormones also moderate stress sensitivity, which in turn influences emotion regulation [38, 41].

Individual women’s vulnerabilities towards the effects of these fluctuations on the brain are located on a continuum [4, 18••]. This requires further risk factors to culminate in a PMD, and probably more specific risk factors for differentiating PMDD, PME of depression, and PME of BD.

### Modulation of Neurotransmitter Systems by Estradiol and Progesterone

Sex hormones E2 and P4, and further neurosteroids such as the progesterone derivative allopregnanolone (ALLO) modulate mood-related brain systems including neurotransmitter synthesis and metabolism, receptor synthesis, and synaptic plasticity [31, 42]. Their cyclic fluctuations result in changes in the activation of limbic and prefrontal brain regions, which are critical to emotion regulation, and can finally induce cyclical changes in mood and behavior [31, 43]. This hormonal priming may have several possible underlying mechanisms on different levels, which have not been fully uncovered [44], but might explain the inconsistencies and individual differences in the menstrual cycle effects on mood symptoms. Possible mechanisms through which sex hormones and their metabolites can regulate brain systems include acute activational effects, programming effects, neural excitability, regulation of neural cell functions and transmission, and circuit regulation [1•].

In particular, the modulating actions of sex hormones on neurotransmitter systems related to mood disorders and their treatment have been implicated in PMDs [6•, 9, 45•, 46]. E2 increases neural excitability by potentiating glutamate release, by suppressing transmission of the inhibitory gamma aminobutyric acid (GABA) neurotransmitter, and by increasing dopamine synthesis and decreasing its degradation and reuptake [45•, 47, 48]. Furthermore, E2 promotes serotonin synthesis and availability, potentiates serotonergic transmission in limbic regions, and also modulates the noradrenergic system by increasing norepinephrine synthesis and availability [49]. Due to the mood stabilizing effects of E2, its fluctuating and decreasing levels during the premenstrual phase and its interaction with the serotonergic and the noradrenergic system have been proposed to contribute to PMDs [25•, 49, 50].

P4 and its metabolite ALLO inhibit glutamate release, and ALLO inhibits dopamine-induced glutamatergic release in the prefrontal cortex, thereby decreasing neuronal excitability. An important role for ALLO is given by its potentiation of the GABAergic synapses. Positive GABA_A_ receptor (GABA_A_R) modulators such as ALLO generally exert sedative, anxiolytic, and antiepileptic effects [51, 52]. Lowered GABA concentrations in the orbitofrontal and the prefrontal cortex have been found in patients with MDD, postpartum depression, and postmenopausal depression [6•], and antidepressant therapy has been linked to an increase in ALLO [6•, 45•]. In animal models, antidepressant effects have been shown by ALLO-induced elevations of the brain-derived neurotropic factor (BDNF).

ALLO has been also implicated in the pathophysiology of PMDD. Together with progesterone, ALLO levels increase after ovulation, reaching its highest levels during the mid-luteal phase, while decreasing during the premenstrual phase. Hantsoo and Epperson [51] propose that women with PMDD show suboptimal sensitivity to the GABA_A_R-enhancing effects of ALLO, thereby provoking premenstrual symptoms and, due to a poor GABA control over the HPA axis, increased subjective and physiological stress sensitivity [53]. The efficacy of selective serotonin reuptake inhibitors (SSRIs) in the treatment of PMDD is thought to reflect their enhancement of ALLO biosynthesis from progesterone, thus stabilizing ALLO levels during the late luteal phase and preventing rapid progesterone withdrawal [51, 54, 55]. Notably, the rapid action of SSRIs, their efficacy in intermittent treatment confined to the luteal phase, and the lower dosing required in PMDD [56] imply different treatment mechanisms of SSRIs in PMDD and MDD [25•, 55]. Another theory poses that due to increased sensitivity of the GABA_A_R to ALLO, ALLO levels during the luteal phase — in a sense of a paradox reaction — induce premenstrual negative mood in women with PMDD [57]. However, blocking the progesterone production reduced premenstrual symptoms in PMDD but not in women with PME of depression [see below; 58].

In addition to premenstrual exacerbations of foremost depressed symptomatology, exacerbations of mainly (hypo-) manic symptoms are observed around ovulation in BD [24•], which points to a further divergent pathophysiological mechanism in BD symptom exacerbation. Elevating E2 levels during the periovulatory phase upregulate the dopaminergic reward system [47, 48], and nonclinical studies indicate increases in rewarding and risky behaviors [59]. Similarly, high E2 levels and increased neuronal excitability may particularly trigger hypomanic and manic episodes during this phase [24•].

### Pharmacokinetics

Sex hormones and their cyclic fluctuations affect physiological systems related to drug processing and are assumed to constitute a main factor for variability in pharmacokinetics and pharmacodynamics of drugs [60]. This also applies to antidepressant treatments, suggesting less stable efficacy and adverse effects in women and contributing to inconsistent results on possible sex differences in response to antidepressant treatment [60]. Women with PME of mood disorders may be particularly vulnerable towards menstrual cycle–related serum level variability of drugs and may therefore need specific drug monitoring and adaption. Systematic research on this topic is lacking for PME of depression, while a recent small-scale review [31] showed a link between PME in (hypo-) manic and psychotic symptoms and a decrease in lithium serum levels during the luteal phase in women with BD. In one case report [61], a higher dose of lithium in the premenstrual phase appeared to compensate the premenstrual decline in serum levels and to prevent PME symptoms.

In particular, treatment of BD with mood stabilizers appears to affect further menstrual cycle–linked abnormalities including hypothalamic-pituitary-gonadal (HPG) axis and metabolic dysfunctions, which interfere with menstrual cycle functioning [62]. Both lithium and valproate facilitate menstrual cycle irregularities [63], and valproate may provoke polycystic ovarian syndrome, thereby causing elevated testosterone levels, anovulation, and infertility [63, 64]. Most studies in BD excluded women with menstrual cycle irregularities, which substantially limits generalization.

## Treatment of PME in Mood Disorders

### Depressive Disorders

Large-scale treatment studies on PME of depression are lacking. Some interventions have built on treatments for PMDD, which involved suppression of ovulation by oral contraceptives (OCs) or by gonadotropin-releasing hormone (GnRH) agonists, and ALLO inhibition. Small case series investigated variable dosages of antidepressants across the menstrual cycle.

#### General Mood Effects of OCs in Women With Depression

Previous reviews have shown conflicting results on whether OCs are beneficial, neutral, or mood worsening in women with mood disorders [3, 65, 66, 67]. Women with a history of depression have shown mood worsening under OCs [68, 69], and OC use was linked to an increased incidence of depression diagnosis, antidepressant treatment, and suicidality, particularly in younger cohorts, in a large case register study [70]. Others did not identify increases in affective symptoms in women with MDD [71, 72]. However, these studies usually did not focus on specific cycle phases and did not distinguish between women with PMDD, PME of depression, and noncyclical depression. Therefore, their informative value for treating PME in depression is limited.

#### Augmentation of Antidepressants with Combined Oral Contraceptives Including Drospirenone

A combination of ethinyl estradiol (EE) and drospirenone, a synthetic progesterone derivate, delivered in a 24/4 dosage regimen, has proven effective for the treatment of PMDD and PMS [73].

Joffe et al. [74] randomized 25 depressed women who were fully remitted on stable doses of antidepressants, but showed premenstrual breakthroughs of depressive symptoms, to EE 30 µg/day plus drospirenone 3 mg/day for 21 days and double-blinded EE 30 µg/day or placebo for days 22 to 28 of two cycles. In both groups, premenstrual depression levels were reduced to postmenstrual levels after 2 months. Since this study missed a true placebo condition, its implications remain unclear. Peters et al. [75] randomized 32 women, who were currently remitted under stable antidepressant dosages but showed a 30% increase in depression scores from the follicular to the late luteal phase, to augmentation with either EE 20 µg/day plus drospirenone 3 mg/day for 24 days or placebo for two cycles. Decreases in depression scores and premenstrual symptom scores were similar in both groups, suggesting that augmentation of antidepressant treatment by OCs containing drospirenone is not effective for treating PME of depression.

#### Suppression of Ovulation by GnRH Agonists

GnRH agonists inducing a reversible menopause, such as leuprolide, are effective in the treatment of PMDD [76]. However, studies on women with PME of depression revealed no beneficial results. Freeman et al. [77] administered 3.75 mg of depot leuprolide monthly in seven women with prospectively assessed PMS and in two women with PME of MDD. In six PMS cases, premenstrual symptoms were reduced to levels of their follicular phase, whereas little improvement in premenstrual symptoms and even a slight increase in depressed symptoms were observed in the two MDD patients. In a second study, Freeman et al. [78] randomized 33 women with PMS or PME to 3.75 mg of depot leuprolide or placebo. Here, only the PMS leuprolide group improved significantly. These studies suggest that leuprolide does not reduce PME in women with ongoing postmenstrual depressive symptoms.

#### GABAA Antagonist Sepranolone (UC1010)

ALLO can be inhibited by the GABA_A_ modulating steroid antagonist Sepranolone (UC1010). Subcutaneously applied UC1010 in women with PMDD [58] significantly improved PMS symptoms and marginally improved core affective PMDD symptoms. Excluding women with elevated symptom levels during the follicular phase at baseline increased the overall treatment effect of UC1010 in the pure PMDD group. This suggests a blunted or non-existing effect of UC1010 in women with PME of depression.^1^ Other substances reducing ALLO activity that have proven effective for PMDD such as dutasteride [79] or ulipristal acetate [80] have so far not been tested in women with PME of depression.

#### Variable Dosing of Antidepressants

A small case report series of PME in unipolar and bipolar depressive patients [81] showed that in some cases, variable dosage augmentation of antidepressants during the luteal phase predicted greater symptom improvement and less switches into (hypo-) manic states together with higher tolerability compared to a continuation with lower dosages, or higher dosages, respectively. In another small case series [82], three women with prospectively validated PME of MDD, who were on constant doses of nefazodone for 2 months, received an augmentation of 50 mg nefazodone or placebo prior to expected menses for 3 months and were crossed over to the other condition thereafter. Each woman showed premenstrual symptom improvement under augmentation compared to placebo. Dosage increase also improved depressive symptoms during the follicular phase.

Another small pilot study [17] investigated effects of variable dosing of sertraline in women with PME of MDD. During the first phase of the study, women rated their daily mood symptoms while being treated with a constant dose of sertraline (50 or 100 mg, depending on the individual patient’s need) for at least 2 months. Those who showed continued PME of symptoms were entered into a crossover design and received placebo or a 50% increased dose of sertraline during the last 10 days of the menstrual cycle for 2 months. Premenstrual depressive symptoms decreased to the levels of the follicular phase during supplemental sertraline, whereas during placebo, the PME pattern was remained stable. Blood sertraline levels paralleled the change in dosing patterns.

#### Psychotherapy

No studies are available on psychotherapeutic approaches for PME of depression.

### Bipolar Disorder

The treatment of BD mainly involves antipsychotics and mood stabilizers such as lithium and antiepileptics (first-line treatments) and adjunctive SSRIs or buproprion (second-line treatments) for acute and maintenance intervention [22]. Systematic treatment research on PME in BD is lacking.

#### Antidepressants

Monotherapies with antidepressants, such as SSRIs, which have been proven effective for PMDD, confer the risk of treatment-induced affective switch into hypo/mania and induction of rapid cycling in women with PME of BD [22, 24•]. Combined treatment of antidepressants with mood stabilizers during bipolar depressive episodes appears not to increase this risk [83, 84], while antidepressants during manic states should be avoided [22]. SSRI treatment has been suggested for BD women who have responded well to mood stabilizers but show premenstrual breakthroughs or comorbid PMDD [85], but these recommendations are not based on empirical evidence.

#### Mood Stabilizers 

Appropriate maintenance treatment in women with PME of BD significantly reduces or prevents mood fluctuations across the menstrual cycle [64]. Karadag et al. [86] showed that euthymic women with BD who were treatment responsive on lithium or valproate showed less prospectively assessed premenstrual symptom increases than healthy controls, indicating their prophylactic effects against PME. Premenstrual mood stabilizing effects are also reported for lamotrigine [87]. Mood stabilizers have been suggested to exert their qualities due to their modulating effects on the glutamatergic and GABAergic system [88]. In women with BD during euthymia, the plasma concentration of ALLO was elevated in the premenstrual period compared to healthy controls and women with MDD, and was therefore assumed to act as an endogenous mood stabilizer [89]. In contrast to PMDD, there is no research to date on the role of GABA_A_ modulating treatments affecting ALLO levels in women with PME of BD [85].

#### Atypical Antipsychotics

Clinical trials investigating the efficacy of atypical antipsychotics for PME of BD are lacking. A case report of a woman with BD-I and premenstrual psychosis and mood instability provides first indications for possible efficacy of atypical antipsychotics such as lurasidone in combination with lithium [90].

#### Hormonal Contraceptives

An understudied but important aspect is the impact of OCs on mood and pharmacokinetics in women with BD [34], which is sometimes recommended as a preferred solution over SSRIs in stabilized women with BD showing PMDD-typical cyclicity [21, 85]. Rasgon et al. [91] showed that women with BD experienced less mood fluctuations across the menstrual cycle when taking OCs, while others identified no impact of OCs on mood [92], or even mood worsening especially with mixed or depressive symptoms [93]. One study reported that OCs may act synergistically with medications which modulate the GABA_A_R activity to enhance mood such as Lamotrigine, but this was based on an exploratory post hoc analysis and needs further systematic investigation [87]. Moreover, opposed interactive effects of mood stabilizers and OCs have been observed [64]: several anticonvulsants used for BD treatment can decrease the effectiveness of OCs, and estrogen-containing OCs, in turn, can decrease lamotrigine and valproate levels [63]. Thus, the exact ingredients of both psychotropic drugs and hormonal contraceptions and their synergic effects must be considered and monitored in order to protect women from unintended pregnancies and maintain therapeutic levels of medications [3, 22, 42, 63, 65].

##### Psychotherapy

No studies are available investigating psychological interventions for PME in BD.

## Conclusions

In conclusion, limited empirical evidence suggests that around 60% of women with depressive disorders and a similar proportion of women with BD experience symptoms of PME. PME predicted nonresponse to treatment, a more chronic or recurrent course of illness, and impaired functioning in both disorders. However, most studies suffer from retrospective assessments of PME, lack of control of hormonal contraceptives and psychotropic drug use, and objective validation of ovulation. Therefore, larger community-based and clinical studies are needed, which use prospective ratings covering the whole spectrum of depressive or BD symptoms and PMDD symptoms, and which provide comprehensible algorithms for the definition of pre- to postmenstrual change scores to specify PME, together with a comprehensive investigation of PME risk factors.

Studies on menstrual cycle–related changes in women with BD suffer from particular methodological flaws. Most studies do not distinguish between PME of BD and BD comorbid with PMDD. Although different subtypes of menstrual cycle effects in BD have been proposed, including entrainment, exacerbation, comorbidity of BD with PMS/PMDD, and magnification, defined as BD with PME and comorbid PMDD/PMS [24•], a clear operationalization of these subtypes is lacking. Also, further relevant cycle phases in BD, particularly the preovulatory phase, are underinvestigated [23, 24•, 37]. Here, larger systematic research is needed to assess frequencies and impact of clearly defined subtypes of menstrual cycle–related phenomena in BD.

In general, comorbidity rates of PMDD in mood disorders may be overestimated. In particular, premenstrual depressive or (hypo-) manic breakthroughs or entrainments in affected women who are treatment-responsive during the other cycle phases should be correctly classified as PME, and not as comorbid PMDD, if the symptoms are part of the ongoing disorder [12, 24•].

With respect to underlying biological mechanisms, it is plausible that cyclical changes in sex hormones and their metabolites during the premenstrual phase, and also during the periovulatory phase in BD, affect the modulation of neurotransmitters and contribute to PME in mood disorders in vulnerable women [24•, 49]. However, the complexity of implicated risk factors and underlying mechanisms has so far not been systematically explored. In particular, there is indication that the pathophysiology of PMDD and PME of depression do not completely overlap. Although ovarian suppression with OCs and GnRH agonists as well as ALLO inhibition appears to be effective in treating PMDD (but see footnote 1), studies do not support their efficacy for PME of depression [58, 74, 75, 77, 78]. In particular, the hypothesized paradoxical reaction towards ALLO [9] is probably specific for PMDD, whereas women with PME of depression might rather be susceptible for progesterone withdrawal during the premenstrual phase [18••]. The role of the GABAergic system is an increasing topic in BD research [88], but its role in the treatment of PME of BD has not yet been studied.

The extent to which PMEs of mood disorders reflect pharmacokinetic effects remains to be determined. Larger studies that systematically assess possible fluctuations of serum levels of antidepressants and mood stabilizer together with fluctuating sex hormone levels and mood symptoms across the cycle are warranted [cf. 31, 60].

Small trials and case reports preliminarily indicate that when cycle-related recurrences of symptoms or side effects occur on constant dosage of antidepressants in PME of depression, periodic dosage increases during the luteal phase could improve efficacy, reduce side effects, and increase compliance, and may be more cost-effective compared to fixed increased schedules during the whole cycle [17]. However, larger RCTs are urgently needed to come to more reliable conclusions regarding the benefits of antidepressant augmentation for PME of depression.

A similar lack of systematic treatment research exists for PME in BD where only small studies and case reports are available indicating that adequate monitoring and adaption of serum levels of mood stabilizers and antipsychotics reduce cyclic variation of mood symptoms [64, 86, 87, 89]. A beneficial effect of OCs in reducing PME in BD has not yet been established, and care should be taken considering possible adverse interaction effects with mood stabilizers [3, 22, 42, 65]. Despite this lack of evidence, treatment guidelines for PMD continue to recommend ovulation suppression for women with PME of mood disorders. For example, the ISPMD [94] recommends that “premenstrual exacerbation of an underlying physical or psychiatric disorder may be treated either by managing the symptoms of the underlying condition… or by ovulation suppression …” (p. 288). Similarly, the Interdisciplinary Expert Meeting on consensus of premenstrual disorders in Switzerland [95] recommend that treatment of PME of ongoing disorders “… should aim to treat underlying medical, physical or psychiatric condition or suppress ovulation (or both)” (p. 347) as first-line treatment.

Finally, while there is clear evidence that psychological treatments improve treatment adherence, symptomatology, and psychosocial functioning in mood disorders, both for unipolar depression [96] and BD [22], no studies have investigated the efficacy of psychological interventions for PME of mood disorders so far. For example, Weise et al. [97] showed promising effects of internet-guided cognitive behavior therapy for PMDD, which could be transferred and tested, possibly more specifically and individually adapted, in women with PME of depression and BD. Other approaches that could be tested in future intervention research include acceptance of symptoms [98], mindfulness approaches, and trainings in self-monitoring and cycle awareness with individually tailored coping skills use [99].

To sum up, a remarkable paucity of research has addressed epidemiology, underlying mechanisms, and treatment options for PME of mood disorders. Given the immense burden for affected women, there is a clear need for increased systematic research on all of these issues together with a more comprehensive consideration of menstrual cycle–related phenomena in mood disorders. For an adequate clinical care management, the role of PME should be included into the training curricula of psychiatrists, gynecologists, and psychologists, as already proposed for PMDD [100].

## Data Availability

Not applicable.

## Footnotes

1 The promising study results for women with pure PMDD could not be confirmed in a subsequent large international multicenter phase IIb study, in which UC1010 was not more effective than placebo, leading to a premature termination of the trial (cf. https://asarinapharma.com/pmdd/sepranolone-and-pmdd/).
